# Physicians Visiting List for 1883

**Published:** 1882-12

**Authors:** 


*The Physicians' Visiting List for* 1883. P. Blakiston, Son, & Co. Publishers, Philadelphia:

For years past we have annually noticed this excellent publication which is as well adapted to the dental practititioner as to the medical, for an engagement book. The present edition contains the usual contents and affords every facility for the daily record of patients. The posological table which is very complete, is of great value, giving the doses of remedies expressed in terms of apothecaries weights and measures together with the metric terms. The French decimal system of weights and measures is fully explained.

